# Network structure of depression and anxiety symptoms in Chinese female nursing students

**DOI:** 10.1186/s12888-021-03276-1

**Published:** 2021-05-31

**Authors:** Lei Ren, Yifei Wang, Lin Wu, Zihan Wei, Long-Biao Cui, Xinyi Wei, Xinyu Hu, Jiaxi Peng, Yinchuan Jin, Fengzhan Li, Qun Yang, Xufeng Liu

**Affiliations:** 1 Department of Military Medical Psychology, Air Force Medical University, Xi’an, 710032 China; 2 Department of Neurology, Xijing Hospital, Air Force Medical University, Xi’an, 710032 China; 3 Department of Psychology, Renmin University of China, Beijing, 100000 China; 4 Department of Psychology, Social Science, University of California, Irvine, California 92614 USA; 5 College of Teachers, Chengdu University, Chengdu, 610106 China

**Keywords:** Depression, Anxiety, Comorbidity, Suicide ideation, Network analysis, Female nursing students

## Abstract

**Background:**

Comorbidity between depressive and anxiety disorders is common. From network perspective, mental disorders arise from direct interactions between symptoms and comorbidity is due to direct interactions between depression and anxiety symptoms. The current study investigates the network structure of depression and anxiety symptoms in Chinese female nursing students and identifies the central and bridge symptoms as well as how other symptoms in present network are related to depression symptom “thoughts of death”.

**Methods:**

To understand the full spectrum of depression and anxiety, we recruited 776 Chinese female nursing students with symptoms of depression and anxiety that span the full range of normal to abnormal. Depression symptoms were measured by Patient Health Questionnaire-9 while anxiety symptoms were measured by Generalized Anxiety Disorder 7-Item Questionnaire. Network analysis was used to construct networks. Specifically, we computed the predictability, expected influence and bridge expected influence for each symptom and showed a flow network of “thoughts of death”.

**Results:**

Nine strongest edges existed in network were from the same disorder. Four were between depression symptoms, like “sleep difficulties” and “fatigue”, and “anhedonia” and “fatigue”. Five were between anxiety symptoms, like “nervousness or anxiety” and “worry too much”, and “restlessness” and “afraid something will happen”. The symptom “fatigue”, “feeling of worthlessness” and “irritable” had the highest expected influence centrality. Results also revealed two bridge symptoms: “depressed or sad mood” and “irritable”. As to “thoughts of death”, the direct relations between it and “psychomotor agitation/retardation” and “feeling of worthlessness” were the strongest direct relations.

**Conclusions:**

The current study highlighted critical central symptoms “fatigue”, “feeling of worthlessness” and “irritable” and critical bridge symptoms “depressed or sad mood” and “irritable”. Particularly, “psychomotor agitation/retardation” and “feeling of worthlessness” were identified as key priorities due to their strongest associations with suicide ideation. Implications for clinical prevention and intervention based on these symptoms are discussed.

**Supplementary Information:**

The online version contains supplementary material available at 10.1186/s12888-021-03276-1.

## Introduction

Depression and anxiety are common mental disorders. A retrospective study of prevalence rates in 44 countries found that the worldwide prevalence of anxiety disorders was estimated at 7.3%, which is equivalent to one in 14 people worldwide suffering from anxiety disorders at any given time [1]. The one-year prevalence of major depressive disorder varies among countries worldwide, but the overall level is about 6% [2]. Comorbidity between depressive and anxiety is common. For example, in the Netherlands Study of Depression and Anxiety, 67% of individuals diagnosed with primary depression had a current comorbid anxiety diagnosis. Similarly, 63% of individuals diagnosed with primary anxiety had a current comorbid depression diagnosis [3]. The level of comorbidities is usually related to the severity of the illness, chronicity, and inability to function well in daily life [4–6]. As we all know, the presence of one mental disorder of depression or anxiety often acts as a trigger for the other [7, 8]. Recently, a meta-analysis study shows that symptoms of depression and anxiety can be predicted from one another during a period of time such as weeks and months [9]. As a result, when symptoms of one disorder are present, the risk of onset of symptoms of the second disorder may rise correspondingly.

In recent years, the network model to psychopathology has been advanced as an alternative way of conceptualizing mental disorders. This model can analyze the relationship between complex variables from a mathematical point of view and display it intuitively [10, 11]. It is data-driven rather than relying on previous assumptions about causality between variables [10, 12]. The network is composed of two parts, one is the node, which represents the variable, and the other is the edge, which represents the relationship between the variables [13]. In this model, mental disorders arise from the direct interactions of different symptoms [14–16]. In other words, mental disorders are emergent phenomena caused by the direct interactions between their corresponding symptoms rather than unobserved latent entities that cause the emergence of symptoms. An accurate description of these interactions is key to interpreting psychopathological mechanisms and developing targeted intervention strategies. Compared with the mere correlational approaches, network model can provide the corresponding centrality and predictability index for each node to examine its importance and controllability in the whole network [17, 18]. Central symptoms in mental disorders may be potential targets for clinical interventions. In addition, network model also provides a new perspective on the understanding of comorbidities [19, 20]. When a person suffers from a certain disorder, the corresponding symptoms of the disorder may increase the risk of other disorders, thus leading to the diagnosis of comorbidities, and the symptoms that increase the risk of other disorders are considered as bridge symptoms [19]. The network model to psychopathology suggests that bridge symptoms may play a role in the development and maintenance of comorbidities. Therefore, it is possible for clinicians to prevent and treat comorbidities from the perspective of bridge symptoms [19, 20].

In the studies of depression and anxiety symptoms, researchers found different and common network characteristics in different samples by applying the network model [10, 21–24] (Wei Z, Ren L, Wang X, Liu C, Cao M, Hu M, et al. Network of depression and anxiety symptoms in patients with epilepsy 2021. (under review).). For example, the relations between “fatigue” and “sleep difficulties”, between “anhedonia” and “depressed or sad mood”, and between “nervousness or anxiety” and “uncontrollable worry” were strong in both migrant Filipino domestic workers and a psychiatric sample [10, 22]. In addition, “depressed or sad mood” and “worry too much” were considered as the most central symptoms in both migrant Filipino domestic workers and a psychiatric sample [10, 22]. “Fatigue” was disclosed as bridge symptom which increases risk of comorbidity between depression and anxiety in migrant Filipino domestic workers [22]. These network studies have provided new insights into the relations and comorbidities of depression and anxiety symptoms. Moreover, network analysis can help us better understand how symptoms related to suicidal ideation. “Thoughts of death” was closely related to “psychomotor agitation/retardation” in migrant Filipino domestic workers [22], while it was related to “depressed or sad mood” and “feeling of worthlessness” in a psychiatric sample [10].

Nursing has historically been a female-dominated profession and a large number of previous studies have shown that the incidence of depression and anxiety is high among nursing students [25, 26]. A previous study, which collected 2111 undergraduate nursing students from various institutions in the United States, showed that 16% of them expressed symptoms consistent with moderate major depressive disorder (MDD), and about 10% of them reported symptoms consistent with moderate generalized anxiety disorder [27]. Unlike other undergraduate majors, nursing students have a heavy study load, including clinical practice, skill exams and lots of other assignments. In addition to the intense study load, they also have to deal with financial burdens, interpersonal problems and other factors that may put them at increased risk of depression, anxiety and suicidal ideation [25]. In addition, previous studies have shown that women are almost twice as likely to suffer from depression and anxiety disorders as men [28, 29]. Suicide is the second most common cause of death among young people aged 15–29, and women have higher rates of suicidal ideation than men [30–32]. For students majoring in nursing, Goetz held opinion that they may have a high risk of suicide compared with college students of other majors, and the reason may be related to the high pressure of academic curriculum for nursing students [33]. A study of nursing students from Greece showed that the incidence of lifetime suicide ideation was about 10.6%. The results also showed that 1.7% female participants reported the thoughts to actually die though suicide if they had an opportunity [34]. Therefore, it is necessary to investigate symptoms of depression and anxiety in female nursing students from a network perspective to provide related clinical implications.

The current study is the first to apply network model to investigate how symptoms of depression and anxiety relate to each other in Chinese female nursing students. We hypothesize that the symptoms network structure, central symptoms, and bridge symptoms in the present study may have similar and specific results when compared with previous network studies. Particularly, we focus on the symptoms that are directly related to “thoughts of death”. We hypothesize that the symptoms “psychomotor agitation/retardation”, “depressed or sad mood” and “feeling of worthlessness” are closely related to “thoughts of death”.

## Methods

### Ethics statement

This study was reviewed and approved by the Independent Ethics Committee of the First Affiliated Hospital of the Fourth Military Medical University (No. KY20182047-F-1). The current study was an online survey through Wenjuanxing (www.wjx.cn) from 21 August 2020 to 25 August 2020. The first part of this online survey mainly included the informed consent. After reading the informed consent, participants can click “I agree” to complete the following survey if they want to further participate in this study. Next, they will complete the following items. Participants were also reminded that the survey was anonymous and personal information would not be disclosed, except for demographic data obtained in the first part.

### Participants

Research has shown that there are important similarities between those whose symptoms meet the criteria for the disease and those who miss the diagnostic cutoff due to fewer and/or less severe symptoms. To understand the full spectrum of depression and anxiety, we recruited participants with symptoms of depression and anxiety that span the full range of normal to abnormal [35]. In this study, WeChat was used for the dissemination of our online survey, mainly drawing on the fact that WeChat is the most popular social media, with 1.15 billion active users in China [36]. A total of 798 Chinese nursing students from three medical universities participated in our study. All of these participants were undergraduate students majoring in nursing in the School of Nursing. And the three medical universities separately are Guangzhou University of Chinese medicine, Guangzhou Technical School, Rizhao Health School. Twenty-two questionnaires were excluded due to their demographic information is incomplete or the respondents are males. At last, a total of 776 questionnaires were obtained.

### Measures

#### Depression symptoms

The Patient Health Questionnaire-9 (PHQ-9) is a reliable and effective self-assessment questionnaire that measures the frequency of depressive symptoms over the past 2 weeks in clinical practice and research [37]. PHQ-9 has 9 items and the score of each item varies from 0 to 3 (point referred to “not at all”, “several days”, “more than half the days”, and “nearly every day”, respectively). The sum of scores ranges from 0 to 27, and the higher the total score, the higher the level of depression severity. The internal consistency of PHQ-9 in this study was excellent (α = 0.93).

#### Anxiety symptoms

The Generalized Anxiety Disorder 7-Item Questionnaire (GAD-7) is a valid and efficient self-report questionnaire for measuring the frequency of symptoms of generalized anxiety disorder (GAD) over the last 2 weeks [38]. GAD-7 has 7 items and each item varies from 0 to 3 (point referred to “not at all”, “several days”, “more than half the days”, and “nearly every day”, respectively). The sum of scores ranges from 0 to 21, and the higher the total score, the higher the level of GAD severity. The internal consistency of GAD-7 in this study was excellent (α = 0.93).

### Network analysis

The network was estimated via Gaussian graphical model [39]. Gaussian graphical model is undirected network, and its edge represents the partial correlation between nodes after controlling for all other nodes in the network. To account for the ordinal nature of the PHQ-9 and GAD-7, the nonparametric Spearman rho correlations were used when estimating the network structure, as recommended by Epskamp and Fried [40]. The regularization of the Gaussian graphical model was conducted via the graphical LASSO (least absolute shrinkage and selection operator) algorithm [41]. In this regularization process, all edges were shrunk, and the edge with small partial correlation was set to zero, thus a more stable and easier to interpret sparse network can be obtained [40, 41]. At the same time, the tuning parameter was set to 0.5, which was a good balance between the sensitivity and specificity of extracting true edge [40, 42]. The visualization of the network was conducted by the Fruchterman-Reingold algorithm, through which nodes with strong and a large number of connections appear near the center of the network, while nodes with weak and a small number of connections walk around the periphery of the network [43]. In the visualized network, the blue edge represents the positive correlation, while the red edge represents the negative correlation. A thicker edge means a stronger correlation between two adjacent nodes. The network was constructed and visualized using the R-package *qgraph* [44].

We used R-package *qgraph* to calculate the expected influence for each node [44]. Compared with the traditional centrality index (e.g., strength centrality), this indicator is more appropriate for the network has both positive and negative edges [45]. Expected influence is defined as the sum of the value of all edges connecting to a specific node. The higher the expected influence, the more important it is in the network. In addition, we used R-package *networktools* to calculate the bridge expected influence for each node and then identified the bridge symptoms [20]. Bridge expected influence is defined as the sum of the value of all edges connecting a specific node with nodes in the other community. Higher bridge expected influence values indicate greater extent for increasing risk of contagion to other communities [20]. In the current network, nodes were divided into two communities in advance: one community includes nine depression symptoms (PHQ-9) and the other community consists of seven anxiety symptoms (GAD-7). To identify bridge symptoms, we conducted a rigorous method with a blind 90th percentile cutoff on the value of bridge expected influence to avoid the confirmation bias that might arise when we interpret bridge centrality statistics. Moreover, we used R-package *mgm* to compute the predictability for each node [18]. Predictability refers to the extent to which the variance of a node can be explained by all of its neighbors. Predictability can reflect the controllability of the network: when the predictability of a node is high, we can control it via its neighboring nodes; when the predictability of a node is low, we can directly intervene on itself or look for other variables out of the network to control it [18, 46].

We used a graphical function “flow” in R-package *qgraph* to identify which symptoms are directly associated with depression symptoms “thoughts of death”. This function places “thoughts of death” to the left and constructs a vertical network that displays which edges are directly or indirectly associated with “thoughts of death”.

The robustness of network was examined through conducting the R-package *bootnet* [47]. First, we evaluated the accuracy of edge weights via computing 95% confidence intervals using a non-parametric bootstrap approach (2000 bootstrap samples). Second, we evaluated the stability of node expected influences and node bridge expected influences via calculating correlation stability coefficient using a case-dropping bootstrap approach. The value of correlation stability coefficient preferably should be above 0.5 and should not be below 0.25 [47]. Third, we conducted bootstrapped difference tests (2000 bootstrap samples and α = 0.05) for edge weights, node expected influences and node bridge expected influences to examine whether two edge weights or two node expected influences or two node bridge expected influences differ significantly from one another.

## Results

### Descriptive statistics

The mean age of these 776 nursing students was 18.87 ± 0.95 years (mean ± SD, range 18–23 years). All of these participants were female, including 94 sole offspring, 682 non-sole offspring; 575 urban residents, 201 rural residents. Participants’ PHQ-9 and GAD-7 scores reflects the full range of symptom severity. Specifically, on the PHQ-9 (M = 3.90, SD = 4.94), a total of 515 participants had minimal depressive symptoms (range = 0–4), 164 had mild depressive symptoms (range = 5–9), 61 had moderate depressive symptoms (range = 10–14), 23 had moderately severe depressive symptoms (range = 15–19), and 13 had severe depressive symptoms (rang = 20–27). On the GAD-7 (M = 3.35, SD = 3.99), a total of 520 participants had minimal anxiety symptoms (range = 0–4), 199 had mild anxiety symptoms (range = 5–9), 41 had moderate anxiety symptoms (range = 10–14), and 16 had severe anxiety symptoms (range = 15–21). Table 1 shows mean scores, standard deviations and abbreviation for each symptom of the PHQ-9 and GAD-7. Table S1 showed the nonparametric Spearman rho correlation matrix of these depressive and anxiety symptoms (in Supplemental Material).

**Table 1:** Mean scores, standard deviations, predictability and abbreviation for each symptom of the PHQ-9 and GAD-7

Symptoms	Abbreviation	M	SD	Pre
Depression symptoms (PHQ-9)				
PHQ-1: Anhedonia	Anhedonia	0.52	0.73	0.59
PHQ-2: Depressed or sad mood	Sad mood	0.47	0.67	0.67
PHQ-3: Sleep difficulties	Sleep	0.54	0.80	0.55
PHQ-4: Fatigue	Fatigue	0.55	0.74	0.71
PHQ-5: Appetite changes	Appetite	0.44	0.73	0.54
PHQ-6: Feeling of worthlessness	Worthless	0.46	0.72	0.67
PHQ-7: Concentration difficulties	Concentration	0.50	0.74	0.62
PHQ-8: Psychomotor agitation/retardation	Motor	0.22	0.51	0.52
PHQ-9: Thoughts of death	Death	0.19	0.51	0.53
Anxiety symptoms (GAD-7)				
GAD-1: Nervousness or anxiety	Nervous	0.63	0.72	0.60
GAD-2: Uncontrollable worry	Control worry	0.47	0.68	0.71
GAD-3: Worry too much	Worry too much	0.61	0.75	0.71
GAD-4: Trouble relaxing	Relax	0.44	0.68	0.67
GAD-5: Restlessness	Restless	0.28	0.55	0.55
GAD-6: Irritable	Irritable	0.59	0.78	0.66
GAD-7: Afraid something will happen	Afraid	0.33	0.59	0.62

*Abbreviations*: *M* mean, *SD* Standard deviation, *Pre* Predictability

### Network structure

**Fig. 1:**
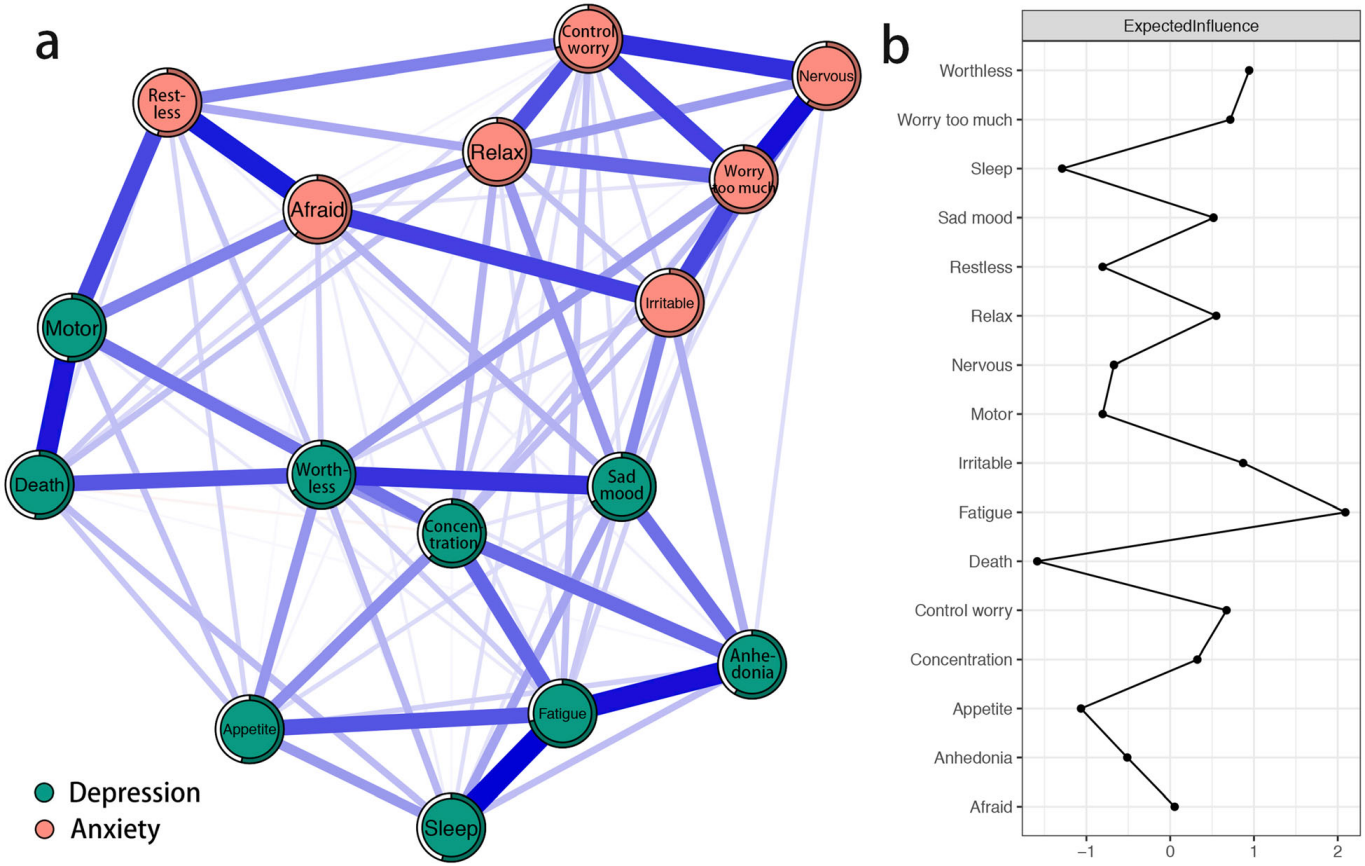
Network structure of depression and anxiety symptoms in Chinese female nursing students. (a) Blue edges represent positive correlations, red edges represent negative correlations. The thickness of the edge reflects the magnitude of the correlation. The circles around nodes depict its predictability. (b) Centrality plot depicting the expected influence of each symptom in the network (z-score)

The network of depression and anxiety symptoms is shown in Fig. 1a. This network shows some characteristics as below. First, 92 edges are not zero (77%) among 120 possible edges and all these edges are positive except the edge between “thoughts of death” and “concentration difficulties” (weight = − 0.01). Second, we find nine strongest edges in the final network. Four of the strongest edges are between depression symptoms “sleep difficulties” and “fatigue” (weight = 0.27), “anhedonia” and “fatigue” (weight = 0.25), “psychomotor agitation/retardation” and “thoughts of death” (weight = 0.24), and “depressed or sad mood” and “feeling of worthlessness” (weight = 0.21). Among anxiety symptoms, we find five strongest are between “nervousness or anxiety” and “worry too much” (weight = 0.25), “restlessness” and “afraid something will happen” (weight = 0.24), “nervousness or anxiety” and “uncontrollable worry” (weight = 0.22), “uncontrollable worry” and “worry too much” (weight = 0.20), and “irritable” and “afraid something will happen” (weight = 0.20). It is noted that those strongest edges have no one which linked anxiety and depression symptoms. Bootstrapped 95% confidence interval indicating the accuracy of edge weights was relatively reliable and accurate (Fig. S1 in the supplementary material). Moreover, in the current network, bootstrapped difference test for edge weights indicates that nine strongest edge weights are significantly different than about 60 to 80% proportion of the other edge weights (Fig. S2 in the supplementary material). Third, node predictability is visualized as circle around node in Fig. 1a. The value of node predictability ranges from 52 to 71%, and the average is 62%. This indicates that on average, 62% of the variance of nodes in the current network can be explained by their neighboring nodes. Depression symptom “fatigue” and anxiety symptoms “uncontrollable worry” and “worry too much” have the highest predictability, indicating that both 71% of their variance can be explained by their neighbors. And depression symptom “psychomotor agitation/retardation” has the lowest predictability, indicating that 52% of its variance can be explained by its neighbors (see Table 1).

The expected influences of depression and anxiety symptoms are shown in Fig. 1b. Depression symptom “fatigue”, “feeling of worthlessness” and anxiety symptom “irritable” have the highest expected influences, indicating that these three symptoms are the most associated symptoms in the current network from the perspective of statistics. Depression symptom “thoughts of death”, “sleep difficulties”, and “appetite changes” have the lowest expected influences, indicating that these three symptoms are the least associated symptoms in the current network from the perspective of statistics. The correlation stability coefficient of node expected influence is 0.67, indicating that the estimations of node expected influences are adequately stable (Fig. S3 in the supplementary material). Moreover, bootstrapped difference tests for node expected influences show that in the current network, the expected influences of three symptoms with highest expected influences are significantly different than about 50 to 90% proportion of the other symptoms (Figs. S4 in the supplementary material).

**Fig. 2:**
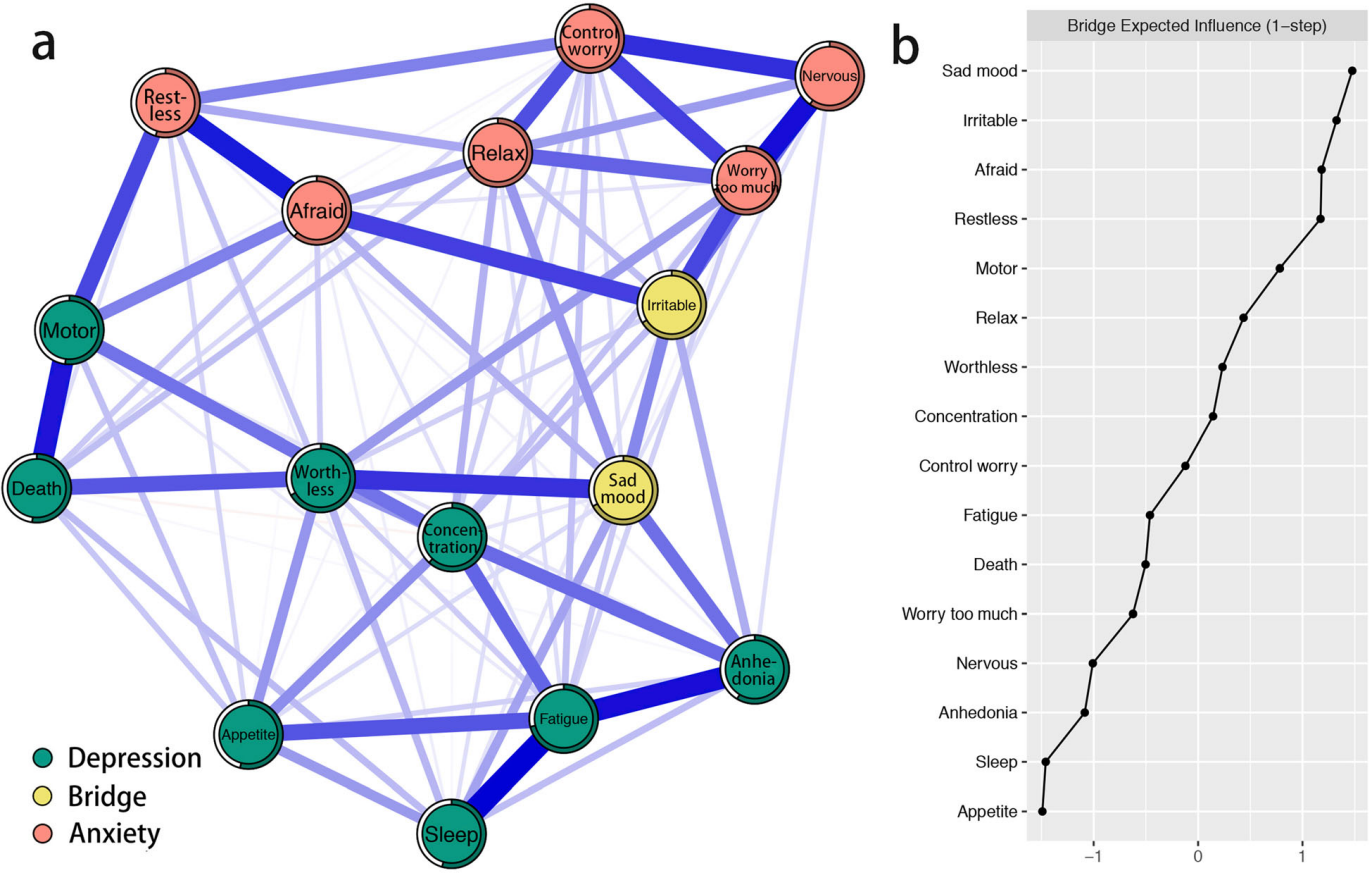
Network structure of depression and anxiety symptoms showing bridge symptoms in Chinese female nursing students. (a) Blue edges represent positive correlations, red edges represent negative correlations. The thickness of the edge reflects the magnitude of the correlation. The circles around nodes depict its predictability. (b) Centrality plot depicting the bridge expected influence of each symptom in the network (z-score)

The bridge symptoms of the depression and anxiety symptom network are shown in Fig. 2a. Based on bridge expected influence in Fig. 2b, results identified PHQ-9 item “depressed or sad mood” and GAD-7 item “irritable” as bridge symptoms. This indicates that “depressed or sad mood” has the strongest ability to increase risk of contagion to anxiety and “irritable” has the strongest ability to increase risk of contagion to depression in the current network. The correlation stability coefficient of node bridge expected influence is 0.52, indicating that the estimations of node bridge expected influences are adequately stable (Fig. S5 in the supplementary material). Moreover, bootstrapped difference tests for node bridge expected influences show that in the current network, the bridge expected influences of bridge symptoms are significantly different than about 50% proportion of the other symptoms (Fig. S6 in the supplementary material).

**Fig. 3:**
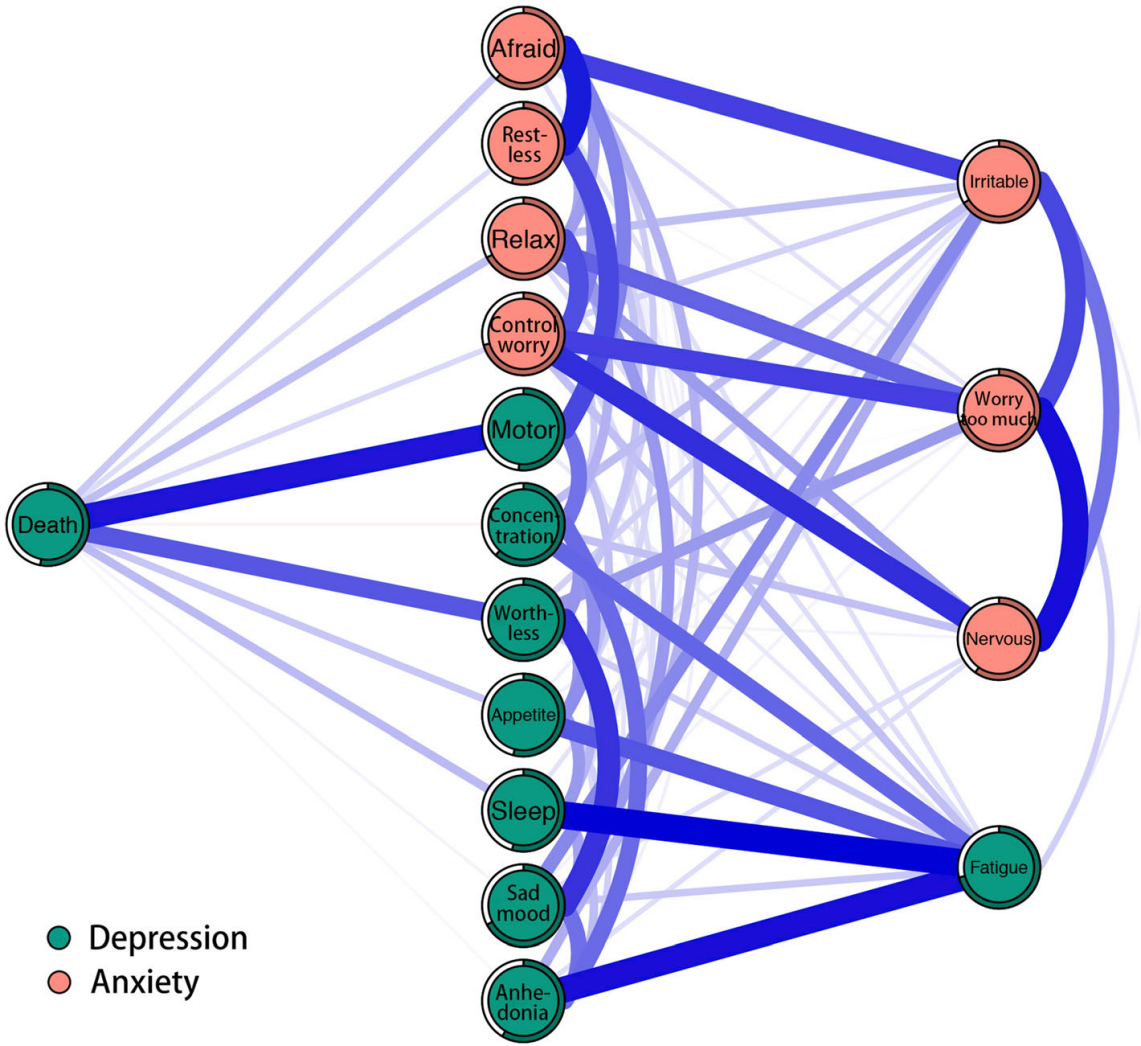
Flow network of suicide thoughts. Blue edges represent positive correlations, red edges represent negative correlations. The thickness of the edge reflects the magnitude of the correlation. The circles around nodes depict its predictability

Figure 3 depicts a flow diagram showing how depression symptom “thoughts of death” is connected to all other symptom of the network. It is obvious that 11 symptoms are directly related to “thoughts of death” and four symptoms are indirectly related to “thoughts of death”. The direct relations between “thoughts of death” and depression symptoms “psychomotor agitation/retardation” and “feeling of worthlessness” are the strongest direct relations. In addition, the predictability of depression symptom “thoughts of death” indicates that 53.00% of its variance can be explained by its neighbors (see Table 1).

## Discussion

This study provides a complex network of depression and anxiety symptoms among Chinese female nursing students. Through this network, we find that the strongest edges exist within each disorder, which is consistent with the results of previous network researches investigating comorbidity of depression and anxiety symptoms [10, 21–24]. Our findings are similar to those of Garabiles et al. and Beard et al. who observed that strongest edges were between “sleep difficulties” and “fatigue”, between “psychomotor agitation/retardation” and “thoughts of death”, between “nervousness or anxiety” and “uncontrollable worry”, and between “uncontrollable worry” and “worry too much” [10, 22].

The current study finds a second strongest edge between “fatigue” and “anhedonia”. To our knowledge, this result has not been discovered in previous studies. This finding may be unique to the specific sample in current study (i.e., Chinese female nursing students), which needs to be further investigated. In fact, according to a recent report on reviewing the similarities and differences between fatigue and anhedonia, approximately 40% of the articles considered fatigue and anhedonia as related or overlapping constructs [48]. Several studies have proposed potential common mechanisms or pathways to elucidate the connection between fatigue and anhedonia. For example, Capuron et al. provided the underlying mechanism by which inflammatory cytokines influence fatigue and anhedonia [49]. In addition, we also find a strong relation between “nervousness or anxiety” and “worry too much”. Indeed, in the fifth edition of Diagnostic and Statistical Manual of Mental Disorders (DSM-5) [50], these two symptoms act as one core symptom of generalized anxiety disorder (i.e., excessive anxiety and worry about various events). The average predictability of present network is 62%, implying that the present network consisted of depression and anxiety symptoms are more likely to be self-determined.

Node expected influence centrality may play an important role in finding symptoms that activate or maintain psychopathological networks as well as providing potential targets for intervention. Depression symptom “fatigue” has the highest centrality which indicates this symptom play the most important role in activating and maintaining psychopathology network of depression and anxiety. Therefore, interventions targeting “fatigue” might generally alleviate both anxiety and depression symptoms in Chinese female nursing students. In addition, this centrality result is consistent with previous studies which investigated symptoms network of depression and anxiety among Filipino immigrant family workers and patients with major depressive disorder [22, 24]. A previous study has shown that moderate to severe fatigue was a very common phenomenon (83.5%) among nursing undergraduate students [51]. And 44 % of the nursing students mentioned that the primary cause of fatigue are related to the characteristics of the nursing course. The excess of activities and sleep disorders are the second and third causes of fatigue [51]. In addition, as the editorial board member suggested, culture may favor physical rather than verbal expression as coping strategy and then leads to fatigue. Researchers have found that Filipinos tend to somaticize to express distress rather than confronting their problems directly [52]. In fact, “fatigue” is an overlapping symptom of major depressive disorder and generalized anxiety disorder, which exist in the DSM-5 diagnostic criteria [50]. The overlapping symptom could explain comorbidity rates. However, the bridge expected influence of “fatigue” is lower than average level of bridge centrality. This may because the edges between “fatigue” and “sleep difficulties”, and “fatigue” and “anhedonia” are the strongest edges. Depression symptom “feeling of worthlessness” and anxiety symptom “irritable” also have high centralities which suggests targeting these symptoms may be efficient to decrease severity of depression and anxiety symptoms in Chinese female nursing students. According to previous studies, irritability is defined as a low threshold for experiencing anger in response to frustration [53, 54]. A recent study showed that symptom “anger” was highlighted the importance to symptomatology in idiographic dynamic network of mood and anxiety symptom [55].

Node bridge expected influence centrality may provide guidance for searching bridge symptoms that play important roles in the development and maintenance of comorbidity of mental disorders. There are two bridge symptoms in the current network, including depression symptom “depressed or sad mood” and anxiety symptom “irritable”. These findings indicate that when depression presents, treating “depressed or sad mood” may decrease risk of contagion to anxiety and when anxiety presents, treating “irritable” may decrease risk of contagion to depression. In previous studies on the bridge symptoms between anxiety and depression, “depressed or sad mood” was also found as a bridge symptom [20, 22]. Further, by conceptualizing mental state of depression and anxiety as a dynamic network, researchers found that “feeling down” was the strongest bridge mental state in both depression-anxiety comorbid and anxiety-only groups [23]. As for “irritable”, it performs as a diagnostic criterion for not only generalized anxiety disorder but also depression in children and adolescents in the DSM-5 [50]. However, the recent research has shown that on mental state level, “irritable” is not relevant as bridge mental states in personal which are prone to suffer from comorbidity of depression and anxiety [23]. This needs to be further investigated.

Even though “thoughts of suicide” ranked the lowest centrality which was quite similar to previous studies [10, 22], it has always been a very important clinical manifestation of depression. In our study, “thoughts of suicide” had the lowest mean and standard deviation, which may have attenuated its expected influence [10]. In addition, what is worth mentioning is that increasing studies present suicide ideation from a network perspective and might provide new insights into this problem [56–59]. In our flow network, “thoughts of death” is directly connected with most of symptoms of depression and anxiety, which to some extent reflects the suicide ideation is actually a complex phenomenon. Our finding that the connection between “thoughts of death” and depression symptoms “psychomotor agitation/retardation” has the strongest correlation coefficient among all direct connections is well aligned with previous study [22]. Among patients with a major depressive episode (MDE), studies have shown that “psychomotor agitation and impulsivity” was one of the most frequently variables related to previous suicide attempts (SA) [60]. The connection between “thoughts of death” and “feeling of worthlessness” also have a great correlation coefficient. Using directed acyclic graphs to explore the relations among symptoms of alcohol use disorder and MDD and suicidal behaviors, researchers found that “worthlessness/guilt” was the symptom directly associated with suicide ideation for both men and women groups [61]. Moreover, “feelings of worthlessness” during MDE was the only symptom that predicted the increase of SA after the remission of MDE [62]. A recent study found that only 3 symptoms from the first 8 PHQ-9 questions (i.e., the second symptom “depressed or sad mood”, the sixth symptom “feeling of worthlessness”, and the eighth symptom “psychomotor agitation/retardation”) were significant explanatory variables for suicidal ideation among adult primary care patients [63]. Previous studies also found that negative beliefs about the self (e.g., low self-worth) is highly prevalent in those who entertain suicidal thoughts [64]. In addition, the relations between “thoughts of death” and “anhedonia” and “depressed or sad mood” are weak which indicate inadequacy of the PHQ-2 (i.e., the first 2 questions of PHQ-9) for identifying Chinese female nursing students with suicide ideations. Overall, these findings suggest that we may have to pay particular attention to any indication that Chinese female nursing students present psychomotor agitation/retardation (i.e., moving or speaking so slowly, or being so fidgety or restless) and feeling of worthlessness (i.e., bad self-feeling, or feel like a failure, or like they have let themselves or their family down). Meanwhile, these findings may also provide several potential pathways for interventions of suicidal ideation. The predictability of “thoughts of death” is 0.53, indicating that “thoughts of death” is moderately affected by its neighboring symptoms in the current network. This result indicates that we could intervene on “thoughts of death” not only through other relevant variables that are not belong to the current network or itself but also by its strong neighboring symptoms (i.e., “psychomotor agitation/retardation” and “feeling of worthlessness”). It is important to note that predictability is the upper bound estimation.

This is the only study to our knowledge to investigate network structure of depression and anxiety symptoms in Chinese female nursing students. The current study provides several possible implications for clinical prevention and intervention to meet the needs of mental health in Chinese female nursing students. First, “fatigue”, “feeling of worthlessness”, and “irritable” have the highest expected influence. From a network perspective, targeting these symptoms might generally alleviate both anxiety and depression symptoms in Chinese female nursing students. Second, we find that “depressed or sad mood” and “irritable” are bridge symptoms. Therefore, depression symptom “depressed or sad mood” may put one at risk of anxiety and anxiety symptom “irritable” may put one at risk of depression. To prevent or to treat comorbidity of depression and anxiety in Chinese female nursing students, bridge symptoms may be the efficient targets. Third, there are strong relations between “thoughts of death” and “psychomotor agitation/retardation” and “feeling of worthlessness”. Through observing and alleviating the “psychomotor agitation/retardation” and “feeling of worthlessness” in Chinese female nursing students, it might be efficient to detect and intervene suicidal ideation in them.

There are some limitations in our study. First, we recruited Chinese female nursing students who majoring in school of nursing and reporting symptoms of depression and anxiety that span the full range of normal to abnormal, which likely limits the generalizability of our findings. For example, depression and anxiety symptoms network in men or clinical sample may be different from the network structure in the current study. In addition, as suggested by a reviewer, the potential influences of depressive disorders, anxiety disorders, and/or other psychiatric disorders of the female nursing students on the estimated network structure have not been evaluated in the present study. These potential influences should be further explored in future studies. Second, the cross-sectional data applied to construct the network structure of depression and anxiety symptoms preclude claims about causality. Therefore, we cannot clarify the causality between the most central symptom and the other symptoms, because there are many possibilities, such as the central symptom activates the other symptoms, or the other symptoms activates the central symptom, or both. Future studies could use intensive longitudinal data to investigate the causality of these symptoms. Third, the network structure constructed here investigated between-subject effects on a group level. This means that within a single individual, the network structure may not be replicated in the same way. Fourth, in this study, the symptoms were single-item, self-reported assessments, which may be limited to capture clinical phenomena. More items and methods could be used in future research. Finally, the network structure in the current study is specific to the questionnaires we used. There are often some differences among self-report tools for assessing symptoms of depression and anxiety. Thus, different self-report tools could result in different network structures.

## Conclusion

In conclusion, this study is the first article investigating network structure of depression and anxiety symptoms in Chinese female nursing students. For centrality indices, “fatigue”, “feeling of worthlessness”, and “irritable” have highest expected influence. Results reveal two bridge symptoms: “depressed or sad mood” and “irritable”. Results also identify “psychomotor agitation/retardation” and “feeling of worthlessness” as critical priority due to they are related to “thoughts of death”. Clinical implications such as treating particular symptoms as targets for preventions and interventions are discussed.

## Supplementary Information

**Additional file 1: Table S1**. Nonparametric Spearman rho correlation matrix of the depression and anxiety symptoms. **Figure S1**. Accuracy of edge weights. **Figure S2**. Bootstrapped difference test for edge weights. **Figure S3**. Stability of node expected influences. **Figure S4**. Bootstrapped difference test for node expected influences. **Figure S5**. Stability of node bridge expected influences. **Figure S6.** Bootstrapped difference test for node bridge expected influences.

## Data Availability

The datasets used and/or analyzed during the current study are available from the corresponding author on reasonable request.

## Abbreviations

MDD: Major Depressive Disorder
PHQ-9: Patient Health Questionnaire-9
GAD-7: Generalized Anxiety Disorder 7-Item Questionnaire
LASSO: Least absolute shrinkage and selection operator
DSM-5: Diagnostic and Statistical Manual of Mental Disorders
M: Mean
SD: Standard deviation
Pre: Predictability
MDE: Major Depressive Episode
SA: Suicide Attempts
