# Supplementary material for: Network structure of depression and anxiety symptoms in Chinese female nursing students
Source: BMC Psychiatry. 2021 May 31;21:279. doi: 10.1186/s12888-021-03276-1 (PMC8168020; doi:10.1186/s12888-021-03276-1)
Supplement: Supplementary file 1 — Additional file 1: Table S1. Nonparametric Spearman rho correlation matrix of the depression and anxiety symptoms. Figure S1. Accuracy of edge weights. Figure S2. Bootstrapped difference test for edge weights. Figure S3. Stability of node expected influences. Figure S4. Bootstrapped difference test for node expected influences. Figure S5. Stability of node bridge expected influences. Figure S6. Bootstrapped difference test for node bridge expected influences. [file 12888_2021_3276_MOESM1_ESM.docx]

**Network structure of depression and anxiety symptoms in Chinese female nursing students**

Lei Ren^1†^, Yifei Wang^1†^, Lin Wu^1†^, Zihan Wei^2^, Long-Biao Cui^1^, Xinyi Wei^3^, Xinyu Hu^4^, Jiaxi Peng^5^, Yinchuan Jin^1^, Fengzhan Li^1^, Qun Yang^1*^, Xufeng Liu^1*^

^1^Department of Military Medical Psychology, Air Force Medical University, 710032, Xi’an, China

^2^Department of Neurology, Xijing Hospital, Air Force Medical University, 710032, Xi’an, China

^3^Department of Psychology, Renmin University of China, 100000, Beijing, China

^4^Department of Psychology, Social Science, University of California, Irvine, 92614, Irvine, California, the United States

^5^College of Teachers, Chengdu University, 610106, Chengdu, China.

^*^ Correspondence: yangqun1125@hotmail.com; lxf_fmmu@163.com

^†^ Lei Ren, Yifei Wang and Lin Wu contributed equally to this work

**Supplementary Materials**

1. Table S1. Nonparametric Spearman rho correlation matrix of the depression and anxiety symptoms
2. Figure S1. Accuracy of edge weights
3. Figure S2. Bootstrapped difference test for edge weights
4. Figure S3. Stability of node expected influences
5. Figure S4. Bootstrapped difference test for node expected influences
6. Figure S5. Stability of node bridge expected influences
7. Figure S6. Bootstrapped difference test for node bridge expected influences

Table S1. Nonparametric Spearman rho correlation matrix of the depression and anxiety symptoms

|  | D1 | D2 | D3 | D4 | D5 | D6 | D7 | D8 | D9 | A1 | A2 | A3 | A4 | A5 | A6 | A7 |
| --- | --- | --- | --- | --- | --- | --- | --- | --- | --- | --- | --- | --- | --- | --- | --- | --- |
| Anhedonia (D1) | 1.00 |  |  |  |  |  |  |  |  |  |  |  |  |  |  |  |
| Sad mood (D2) | 0.64^*^ | 1.00 |  |  |  |  |  |  |  |  |  |  |  |  |  |  |
| Sleep (D3) | 0.58^*^ | 0.59^*^ | 1.00 |  |  |  |  |  |  |  |  |  |  |  |  |  |
| Fatigue (D4) | 0.71^*^ | 0.66^*^ | 0.68^*^ | 1.00 |  |  |  |  |  |  |  |  |  |  |  |  |
| Appetite (D5) | 0.57^*^ | 0.57^*^ | 0.57^*^ | 0.65^*^ | 1.00 |  |  |  |  |  |  |  |  |  |  |  |
| Worthless (D6) | 0.60^*^ | 0.69^*^ | 0.59^*^ | 0.65^*^ | 0.60^*^ | 1.00 |  |  |  |  |  |  |  |  |  |  |
| Concentration (D7) | 0.65^*^ | 0.62^*^ | 0.56^*^ | 0.69^*^ | 0.60^*^ | 0.64^*^ | 1.00 |  |  |  |  |  |  |  |  |  |
| Motor (D8) | 0.50^*^ | 0.50^*^ | 0.44^*^ | 0.53^*^ | 0.50^*^ | 0.53^*^ | 0.56^*^ | 1.00 |  |  |  |  |  |  |  |  |
| Death (D9) | 0.47^*^ | 0.51^*^ | 0.48^*^ | 0.49^*^ | 0.49^*^ | 0.57^*^ | 0.45^*^ | 0.56^*^ | 1.00 |  |  |  |  |  |  |  |
| Nervous (A1) | 0.54^*^ | 0.59^*^ | 0.47^*^ | 0.57^*^ | 0.47^*^ | 0.57^*^ | 0.58^*^ | 0.43^*^ | 0.42^*^ | 1.00 |  |  |  |  |  |  |
| Control worry (A2) | 0.56^*^ | 0.61^*^ | 0.53^*^ | 0.62^*^ | 0.50^*^ | 0.59^*^ | 0.60^*^ | 0.51^*^ | 0.50^*^ | 0.69^*^ | 1.00 |  |  |  |  |  |
| Worry too much (A3) | 0.56^*^ | 0.62^*^ | 0.53^*^ | 0.62^*^ | 0.53^*^ | 0.63^*^ | 0.60^*^ | 0.45^*^ | 0.45^*^ | 0.71^*^ | 0.71^*^ | 1.00 |  |  |  |  |
| Relax (A4) | 0.56^*^ | 0.64^*^ | 0.53^*^ | 0.62^*^ | 0.53^*^ | 0.60^*^ | 0.61^*^ | 0.50^*^ | 0.51^*^ | 0.65^*^ | 0.69^*^ | 0.69^*^ | 1.00 |  |  |  |
| Restless (A5) | 0.50^*^ | 0.54^*^ | 0.47^*^ | 0.50^*^ | 0.50^*^ | 0.55^*^ | 0.52^*^ | 0.57^*^ | 0.49^*^ | 0.50^*^ | 0.58^*^ | 0.54^*^ | 0.58^*^ | 1.00 |  |  |
| Irritable (A6) | 0.61^*^ | 0.66^*^ | 0.55^*^ | 0.63^*^ | 0.55^*^ | 0.63^*^ | 0.63^*^ | 0.50^*^ | 0.46^*^ | 0.66^*^ | 0.65^*^ | 0.71^*^ | 0.66^*^ | 0.55^*^ | 1.00 |  |
| Afraid (A7) | 0.51^*^ | 0.61^*^ | 0.51^*^ | 0.58^*^ | 0.51^*^ | 0.59^*^ | 0.55^*^ | 0.57^*^ | 0.51^*^ | 0.54^*^ | 0.58^*^ | 0.59^*^ | 0.62^*^ | 0.63^*^ | 0.65^*^ | 1.00 |

^*^ *p* < 0.01 (two-tailed)

Figure S1. Accuracy of edge weights

*Note*: The red line depicts the sample edge weights and the gray bar depicts the bootstrapped confidence interval.

Figure S2. Bootstrapped difference test for edge weights

*Note*: Gray boxes indicate edge weights that do not differ significantly from one another, while black boxes indicate edge weights that do differ significantly. Blue and red boxes on the diagonal correspond to edge weights with positive and negative correlations, respectively.

Figure S3. Stability of node expected influences

*Note*: The red bar represents the average correlation between node expected influences in the full sample and subsample with the red area depicting the 2.5th quantile to the 97.5th quantile.

Figure S4. Bootstrapped difference test for node expected influences

*Note*: Gray boxes indicate node expected influences that do not differ significantly from one another, while black boxes indicate node expected influences that do differ significantly. The number in the white boxes (i.e., diagonal line) represent the value of node expected influences.

Figure S5. Stability of node bridge expected influences

*Note*: The red bar represents the average correlation between node bridge expected influences in the full sample and subsample with the red area depicting the 2.5th quantile to the 97.5th quantile.

Figure S6. Bootstrapped difference test for node bridge expected influences

*Note*: Gray boxes indicate node bridge expected influences that do not differ significantly from one another, while black boxes indicate node bridge expected influences that do differ significantly.
